# Visibility Graph Power Geometric Aggregation Operator and Its Application in Water, Energy and Food Efficiency Evaluation

**DOI:** 10.3390/ijerph17113891

**Published:** 2020-05-30

**Authors:** Lihua Liu, Jing Huang, Huimin Wang

**Affiliations:** Business School, Hohai University, Nanjing 211100, China; j_huang@hhu.edu.cn (J.H.); hmwang@hhu.edu.cn (H.W.)

**Keywords:** visibility graph, power geometric operator, visibility graph power geometric operator, water, energy and food efficiency evaluation

## Abstract

In the real decision-making process, there are so many time series values that need to be aggregated. In this paper, a visibility graph power geometric (VGPG) aggregation operator is developed, which is based on the complex network and power geometric operator. Time series data are converted into a visibility graph. A visibility matrix is developed to denote the links among different time series values. A new support function based on the distance of two values are proposed to measure the support degree of each other when the two time series values have visibility. The VGPG operator considers not only the relationship but also the similarity degree between two values. Meanwhile, some properties of the VGPG operator are also investigated. Finally, a case study for water, energy, and food coupling efficiency evaluation in China is illustrated to show the effectiveness of the proposed operator. Comparative analysis with the existing research is also offered to show the advantages of the proposed method.

## 1. Introduction

Information aggregation is a process of fusing a set of values into a single one, which is an important step in selecting the best alternative. Numerous aggregation operators have been proposed over the past few decades. The mostly used aggregation operators are the weighted average (WA) operator [1] and the weighted geometric (WG) operator. Based on these two operators, they are extended to various forms, such as the ordered weighted average (OWA) operator [2], ordered weighted geometric (OWG) operator [3,4], harmonic mean operator [5], induced OWA operator [6], and generalized OWA operator [7]. All of these operators are also extended to various environments, and have generated various operators such as the uncertain OWA operator [8], linguistic aggregation operators [9,10,11], intuitionistic fuzzy aggregation operators [12], hesitant aggregation operators [3], etc.

Among these operators, the most well-known aggregation operator is OWA operator, which is proposed by Yager [2]. The key issue of the OWA operator is to determine its associated weights. A lot of research has been done to obtain the weights. Yager [2] proposed the linguistic quantifier, orness measure, and dispersion measure. O’Hagan [13] developed a procedure to generate the weights, which have a predefined degree of orness, and maximized the entropy of the OWA weights. The procedure can be solved by the Lagrange multiplier [14]. Filev and Yager [15] introduced exponential OWA operators to generate OWA weights satisfying a given degree of orness. Xu [16] developed a normal distribution to determine the OWA weights.

The above proposed operators are usually used for multiple attribute decision making problems. As far as we know, in real situations, there are many time series problems, such as weather forecast, stock, import trade, and so on. It is obvious that the time factor should be also considered. However, the exiting operators fail to consider this face. Further, the proposed method to obtain weights is always objective, as there is no relationship between the values and the weights. Generally, the time series data are relevant before and after. Thus, it is very important to develop an aggregation method that considers the time series values and relationships of these data at the same time. In order to overcome the drawbacks of the existing methods, we develop a new aggregation operator, which is based on the visibility graph (VG) and power geometric (PG) aggregation operator.

VG is proposed by Lacasa et al. [17], which is a powerful tool used to convert a time series to a network. The time series data are plotted by using the vertical bars. If the top of two bars can be linked without interruption by the middle bars, then the two bars are called “visibility.” We develop a visibility matrix to denote the links of the time series data. The visibility only denotes whether the two values are linked, but their similarities are not considered. The power average (PA) aggregation operator is proposed by Yager [18] and is extended to the power geometric (PG) aggregation operator (Xu and Yager [19]) and other types [20,21,22,23,24,25]. The PA operator uses the support function to consider the similarity of two values, and then the similarities between one argument and all the other values are obtained, which is considered as the weight of the argument. However, the weight of each argument depends support of all the other arguments. In this paper, we integrate the above two cases, which consider not only the links, but also the similarity. Based on the VG, we only consider those arguments that are linked with the argument, and the similarities of these linked data are computed, which is more objective and reasonable in the time series.

There are some challenges for the time series aggregation problem that need to be tackled further:

(1) How to determine the weights of the time series values and that the weights are obtained in a more objective way?
(2) How to consider the relationships of in time series values?

The aim of the paper is to tackle these problems. The contributions of the paper are:

(1) A new aggregation operator, namely the visibility graph power geometric (VGPG) aggregation operator, is proposed to aggregate the time series values.
(2) A new support function is proposed to denote the support of the two values when they have visibility.
(3) Based on the support function and visibility matrix, a new objective weight determination method for the VGPG operator is developed.

The reminders of the paper are organized as follows. Section 2 introduces some basic concepts of the OWA operator, PA operator, PG operator, and VG. Section 3 develops a new operator called VGPG aggregation operator. Some properties of the VGPG are investigated. In Section 4, a case study about water, energy, and food efficiency evaluation in China is illustrated to show the effectiveness of the proposed method. Further, the comparative analysis with the existing operators are offered to show the advantages of the proposed method. Finally, the conclusions are given in Section 5.

## 2. Preliminaries

This section introduces the OWA operator, PA operator, PG operator, VG, and VGPA operator. 

### 2.1. The OWA, PA, and PG Operators

Yager [2] first introduced the OWA operator.

**Definition 1.**  *An OWA operator of dimension n is a mapping:F:R^n^→R, that has an associated weighting vectorw = (w*_1_,*w*_2_,…,*w_n_)^T^, such that*$\sum_{i=1}^{n}w_{i}=1$

*, w_i_*∈ [0,1]*, and*

(1) $${\mathrm{OWA}(a}_{1}{,a}_{2},\ldots {,a}_{n})=\overset{n}{\underset{i=1}{\sum }}w_{i}a_{\sigma (i)}$$

*where* *a_δ(i)_* *is the ith largest value of the collection* {*a*_1_,*a*_2_,…,*a_n_*}.

The key issue of the OWA operator is to determine its weights. At present, there are many methods to obtain the OWA weights [16], such as the linguistic quantifiers [2], orness measure, and dispersion measure. O’Hagan [13] used the two measures to generate the OWA weights that have maximized the entropy and a have predefined degree of orness. It needs to solve the following constrained nonlinear optimization method:

(2) $$\max\;\mathrm{Disp}(w)=-\overset{n}{\underset{i=1}{\sum }}w_{i}{\ln(w}_{i})$$

$$s.t.\;\{\begin{array}{c} \mathrm{orness}(w)=\frac{1}{n-1}\overset{n}{\underset{i=1}{\sum }}{(n-i)w}_{i}=\alpha ,0\leq \alpha \leq 1\\ \overset{n}{\underset{i=1}{\sum }}w_{i}=1,\;0\leq w_{i}\leq 1,\;i=1,2,\ldots ,n \end{array}$$

The problem (2) was further investigated by Fullér and Majlender [14].

Yager [18] introduced the PA operator. It is a nonlinear weighted-average operator, which can be defined as follows.

**Definition 2.**  *Let* {*a*_1_,*a*_2_,…,*a_n_*} *be a collection of data, the PA operator is defined as:*

(3) $${\mathrm{PA}(a}_{1}{,a}_{2},\ldots {,a}_{n})=\frac{\sum_{i=1}^{n}{(1+T(a}_{i}{)a}_{i}}{\sum_{i=1}^{n}{(1+T(a}_{i})}$$

*where*

(4) $$T(a_{i})=\sum_{\begin{array}{l} j=1\\ j\neq i \end{array}}^{n}\mathrm{Sup}(a_{i},a_{j}),$$

*and Sup*(*a_i_*,*b_j_*) *is the support for* *a_i_* *from* *a_j_; thus,* *T*(*a_i_*) *is the total support for* *a_i_* *from all the other values except itself. Further, the support function Sup(a*,*b*) *should satisfy the following properties:*

*(1)*  *Sup(a*,*b*) ∈ *[0,1];*
*(2)*  *Sup(a*,*b) = Sup(b*,*a);*
*(3)*  *Sup(a*,*b*) ≥ *Sup(x*,*y), if |a* − *b|<|x* − *y|.*

The property (1) denotes that the support of any two values is between 0 and 1. Property (2) indicates that support between any two values *a* and *b* is equal. Property (3) denotes that if the distance of any two values *a* and *b* is smaller than the other two values *x* and *y*, then the support of *a* and *b* is larger. The support is a similarity function; the closer the two values, the more they support each other.

Based on the PA operator and the geometric mean, Xu and Yager [19] defined the power geometric (PG) operator.

**Definition 3.**  *Let {a*_1_,*a*_2_,…,*a_n_} be a collection of data, the PG operator is defined as:*

(5) $$\mathrm{PG}(a_{1},a_{2},\ldots ,a_{n})=\prod_{i=1}^{n}a_{i}^{\frac{1+T(a_{i})}{\sum_{i=1}^{n}\left(1+T(a_{i})\right)}}$$

*where {a*_1_,*a*_2_,…,*a_n_} is a collection of arguments, andT*(*a_i_*) *satisfies Equation (4). Obviously, the PG operator is a nonlinear weighted-geometric aggregation operator.*

### 2.2. The Visibility Graph

**Figure 1:**
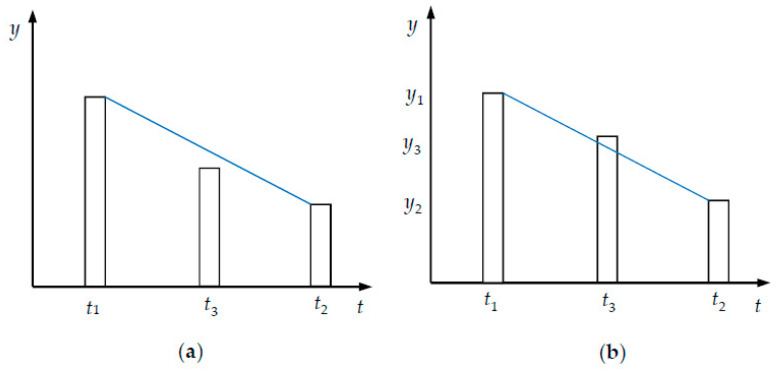
The explanation of VG. The explanation of VG. (**a**) Thus, at the time *t*_3_ between *t*_1_ and *t*_2_ (i.e., *t*_1_ < *t*_3_ < *t*_2_), if the value *y*_3_ is smaller than $\frac{\left(y_{2}-y_{1}\right)}{\left(t_{2}-t_{1}\right)}t_{3}+\frac{y_{1}t_{2}-y_{2}t_{1}}{t_{2}-t_{1}}=y_{2}+\frac{\left(y_{2}-y_{1})(t_{3}-t_{2}\right)}{t_{2}-t_{1}}$, then the top at times *t*_1_ and *t*_2_ can be linked without interruption; otherwise, they cannot be linked; (**b**) *y*_2_ (at time *t*_2_) from *y*_1_ (at time *t*_1_) can not be seen.

The VG method was first proposed by Lacasa et al. [17], and it can convert a time series into a graph. Since its appearance, it has received great attention [26,27,28,29]. In the VG method, the time series is transformed into a network topology. The properties of the time series are conserved in the graph topology. The values of the time series are plotted by using vertical bars. For example, in Figure 1, there are three time series (*t*_1_,*y*_1_), (*t*_2_,*y*_2_), and (*t*_3_,*y*_3_). At time *t*_1_, the value is *y*_1_, at time *t*_2_, the value is *y*_2_, and at time *t*_3_, the value is *y*_3_. Thus, we can plot the time series by the vertical bars. The horizontal is time, and vertical is the value. The line slope between two tops at times *t*_1_ and *t*_2_ is (*y*_2_ − *y*_1_)/(*t*_2_ − *t*_1_), and the linear equation is: *y* =$\frac{{(y}_{2}-y_{1})}{{(t}_{2}-t_{1})}t+\frac{y_{1}t_{2}-y_{2}t_{1}}{t_{2}-t_{1}}$. Thus, at the time *t*_3_ between *t*_1_ and *t*_2_ (i.e., *t*_1_ < *t*_3_ < *t*_2_), if the value *y*_3_ is smaller than $\frac{{(y}_{2}-y_{1})}{{(t}_{2}-t_{1})}t_{3}+\frac{y_{1}t_{2}-y_{2}t_{1}}{t_{2}-t_{1}}=y_{2}+\frac{\left(y_{2}-y_{1}\right)\left(t_{3}-t_{2}\right)}{t_{2}{-t}_{1}}$, then the top at times *t*_1_ and *t*_2_ (See Figure 1a) can be linked without interruption; otherwise, they cannot be linked. That is, we cannot see *y*_2_ (at time *t*_2_) from *y*_1_ (at time *t*_1_) (See Figure 1b). That is to say, if there is a straight line that connects two series data, this “visibility line” does not intersect any intermediate data height. This is the visibility criteria. Thus, the visibility is defined as follows.

**Definition 4**  [17]**.** *Two time series data* (*t*_1_,*y*_1_) and (*t*_2_,*y*_2_) *have visibility for any other data* (*t*_3_,*y*_3_) *such that* *t*_1_ < *t*_3_ < *t*_2_ *satisfies:*

(6) $$y_{3}<y_{2}+(y_{1}-y_{2})\frac{t_{2}-t_{3}}{t_{2}-t_{1}}$$

**Figure 2:**
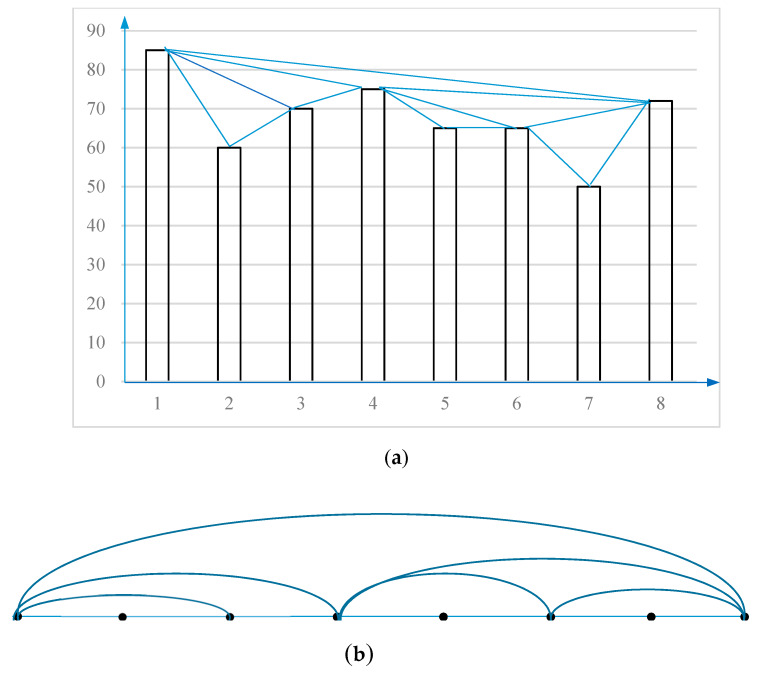
The visibility graph of Example 1. (**a**) the histogram shows a time series with eight data values; (**b**) The visibility rays in the histogram define the links connecting the nodes in the graph.

**Example 1.**  *Assume there is a time series of eight values {85,60,70,75,65,65,50,72} from time t_1_ to t_8_, and the associated visibility graph is shown in Figure 2. In Figure 2a, the histogram shows a time series with eight data values. The first bar links the second, the third, the fourth, and the eighth bars because the tops of these bars can be seen from the first bar. Other links can be explained in the same way. Figure 2b is obtained by Equation (6), which is the topology of Figure 2a, and is called an associated graph. In the graph, each node corresponds to series data in the same order. The visibility rays in the histogram of Figure 2a define the links connecting the nodes in the graph of Figure 2b.*

The associated graph extracted from a time series has the following properties:

(1) Connected: each node connects with its nearest left and right neighbor nodes.
(2) Undirected: there is no direction defined in the links.
(3) Invariant under affine transformations of the series data: the visibility criteria is invariant under the rescaling of both the horizontal and vertical axes.

### 2.3. Visibility Graph Power Averaging Operator

Based on the PA operator and VG, Jiang et al. [30] proposed the visibility graph power averaging (VGPA) operator, and it is defined as follows:

**Definition 5**  [30]**.** *VGPA operator is a mapping VPGA:* *I^n^*→*I, I*∈*R*, *where*

(7) $$\mathrm{VGPA}(a_{1},a_{2},\ldots ,a_{n})=\frac{\sum_{i=1}^{n}(1+T(a_{i}))a_{i}}{\sum_{i=1}^{n}\left(1+T(a_{i})\right)},$$

*where* *a_i_* *is the ith value in the time series, and where*

(8) $$T(a_{i})=\sum_{\begin{array}{l} j=1\\ j\neq i \end{array}}^{n}\mathrm{Sup}(a_{i},a_{j}).$$

*Jiang et al. [30] used the distribution to reflect the important part of a vertex, and defined the Sup(a_i_*,*a_j_) in the VG as:*

(9) $$\mathrm{Sup}(a_{i},a_{j})=\frac{1}{d_{ij}^{2}}.$$

*Thus,*

(10) $$T(a_{i})=\sum_{\begin{array}{l} j=1\\ j\neq i \end{array}}^{n}\mathrm{Sup}(a_{i},a_{j})=\sum_{\begin{array}{l} j=1\\ j\neq i \end{array}}^{n}\frac{1}{d_{ij}^{2}}$$

*whered_ij_* = |*i*−*j| is the distribution from time* *t_i_* *to* *t_j_, and a_i_ and a_j_ are connected.*

**Remark 1.**  *It should be noted that Equation (10) does not have the property of support. For the data in Example 1,a_1_* = 85*, a_3_* = 70*, a_7_* = 50*, and a_8_* = 72*;* *we know that |a*_1_ − *a*_3_| = 15 < |*a*_7_ − *a*_8_| = 22, *but* Sup(*a*_1_,*a*_3_) = 1/2^2^ = 1/4 *and* Sup(*a*_7_,*a*_8_) = 1/1^2^ = 1 *do not satisfy property 3. Thus, a new support function for the visibility graph needs to be designed.*

## 3. Visibility Graph Power Geometric Operator

Based on the VG and PG operator, in the following, we present a new operator called a visibility graph power geometric (VGPG) operator.

**Definition 6.**  VGPG *operator is a mapping* VGPG*: I^n^*→*I*, *I*∈*R*,

(11) $$\mathrm{VGPG}(a_{1},a_{2},\ldots ,a_{n})=\prod_{i=1}^{n}a_{i}^{\frac{1+T(a_{i})}{\sum_{i=1}^{n}\left(1+T(a_{i})\right)}}$$

*where* {*a*_1_,*a*_2_,…,*a_n_*} *can be a time series, and* *T*(*a_i_*) *satisfies Equation (8).*

Obviously, the VGPG operator is a nonlinear weighted-geometric aggregation operator. As *T*(*a_i_*) is the support of the argument *a_i_* from all the other time series values *a_j_* (*j* = 1,…,*n*, *j* ≠ *i*). The weight (1 + *T*(*a_i_*))/$\sum_{i=1}^{n}\left(1+T(a_{i})\right)$ of the argument *a_i_* depends on all the other input series *a_j_* (*j* = 1,…,*n*, *j* ≠ *i*), which can support each other in the geometric aggregation process. As we have pointed out in Remark 1, Equations (9) and (10) do not have the property of support function. Thus, we need to design another way to compute the weight. From the VG theory, if two nodes are connected, it can be regarded that the two nodes are supported each other. By Equation (6), we can develop a visibility matrix

*V* = (*v_ij_*)_*n*×*n*_ (12)

where *v_ij_* = 1 if two values (*t_i_*,*a_i_*) and (*t_j_*,*a_j_*) have visibility (i.e., there is a link from *a_i_* to *a_j_*); otherwise, *v_ij_* = 0. Clearly, *V* is symmetric matrix. Thus, we define the support

(13) $$\mathrm{Sup}(a_{i},a_{j})=\left\{ \begin{array}{l} 1-d(a_{i},a_{j}),\;\;v_{ij}=1\\ 0,\;\;\;\;\;\;\;\;\;\;\;\;\;\;\;\;\;\;\;\;v_{ij}=0 \end{array}\right.$$

where

*d*(*a_i_*,*a_j_*) = |*a_i_* − *a_j_*|. (14)

Here, we assume that *a_i_* ∈ [0,1]; otherwise, it needs to be normalized into the scope [0,1]. Clearly, Equation (13) satisfies the properties of the support function. All the support values obtained by Equation (13) can form a support matrix Sup = (Sup(*a_i_*,*a_j_*))*_n_*_×*n*_.

Furthermore, we denote the following:

*γ_I_* = 1 + *T*(*a_i_*) (15)

and

(16) $$w_{i}=\frac{\gamma_{i}}{\sum_{i=1}^{n}\gamma_{i}}.$$

Obviously, *w_i_* ≥ 0 and $\sum_{i=1}^{n}w_{i}=1$ can be looked at as the weight of *a_i_*. Then, VGPG can be rewritten as:

(17) $$\mathrm{VGPG}(a_{1},a_{2},\ldots ,a_{n})=\prod_{i=1}^{n}a_{i}^{w_{i}},$$

which is a geometric mean aggregation operator. 

In the following, we investigate some properties of the proposed VGPG operator.

**Theorem 1.**  *Letting* Sup(*a_i_*,*a_j_*) = *k* *for all* *i ≠ j, then*

(18) $$\mathrm{VGPG}(a_{1},a_{2},\ldots ,a_{n})=\prod_{i=1}^{n}{\left(a_{i}\right)}^{1/n},$$

*which indicates that when all the supports are the same, the VGPG operator is reduced to a simple geometric averaging operator.*

**Proof.**  If Sup(*a_i_*,*a_j_*) = *k*, and for all *i* ≠ *j*, then

*T*(*a_i_*) = (*n* − 1)*k*. (19)

Thus,

$$\mathrm{VGPG}(a_{1},a_{2},\ldots ,a_{n})=\prod_{i=1}^{n}a_{i}^{\frac{1+(n-1)k}{\sum_{i=1}^{n}\left(1+(n-1)k\right)}}=\prod_{i=1}^{n}a_{i}^{\frac{1+(n-1)k}{n(1+(n-1)k)}}=\prod_{i=1}^{n}{\left(a_{i}\right)}^{1/n},$$

which is a simple geometric averaging operator. □

**Theorem 2.**  (*Boundness*)

$$\underset{i}{\min}(a_{i})\leq \mathrm{VGPG}(a_{1},a_{2},\ldots ,a_{n})\leq \underset{i}{\max}(a_{i}).$$

**Proof.**  Since $\underset{i}{\min}{(a}_{i})\;\leq a_{i\;}\leq \underset{i}{\;\max}{(a}_{i}),$ then

$$\prod_{i=1}^{n}{\left(\underset{i}{\min}a_{i}\right)}^{\frac{1+T(a_{i})}{\sum_{i=1}^{n}\left(1+T(a_{i})\right)}}\leq \prod_{i=1}^{n}a_{i}^{\frac{1+T(a_{i})}{\sum_{i=1}^{n}\left(1+T(a_{i})\right)}}\leq \prod_{i=1}^{n}{\left(\underset{i}{\max}a_{i}\right)}^{\frac{1+T(a_{i})}{\sum_{i=1}^{n}\left(1+T(a_{i})\right)}}.$$

That is:

(20) $$\underset{i}{\min}(a_{i})\leq \mathrm{VGPG}(a_{1},a_{2},\ldots ,a_{n})\leq \underset{i}{\max}(a_{i}).$$

 □

**Theorem 3.**  (*Idempotency*): *If* *a_i_* = *a* *for all* *i =* 1,2,…,*n, then*

VGPG (*a*_1_,*a*_2_,…,*a_n_*) = *a*.

**Proof.**  According to the property of Boundness, we have

*a* ≤ VGPG(*a*_1_,*a*_2_,…,*a_n_*) ≤ *a*.

 □

**Theorem 4.**  VGPG (*ra*_1_,*ra*_2_,…,*ra_n_*) = *r*VGPG(*a*_1_,*a*_2_,…,*a_n_*)*, where* *r* > 0*.*

**Proof.**  *ra*_1_,*ra*_2_,…,*ra_n_* also satisfy Equation (6); therefore, the visibility matrices of *a*_1_,*a*_2_,…,*a_n_* and *ra*_1_,*ra*_2_,…,*ra_n_* are the same. By Equations (13) and (14), we have

Sup(*a_i_*,*a_j_*) = Sup(*ra_i_*,*ra_j_*)

and

*T*(*a_i_*) = *T*(*ra_i_*).

Thus,

$$\mathrm{VGPG}(ra_{1},ra_{2},\ldots ,ra_{n})=\prod_{i=1}^{n}{\left(ra_{i}\right)}^{\frac{1+T(a_{i})}{\sum_{i=1}^{n}\left(1+T(a_{i})\right)}}=r\prod_{i=1}^{n}a_{i}^{\frac{1+T(a_{i})}{\sum_{i=1}^{n}\left(1+T(a_{i})\right)}}=r\mathrm{VGPG}(a_{1},a_{2},\ldots ,a_{n}),$$

which completes the proof of Theorem 4. □

**Lemma 1.**  *Letx_j_* > 0, *λ_j_* > 0*, j =* 1,2*,…, n, and* $\sum_{j=1}^{n}\lambda_{j}=1$ *, then*

(21) $$\prod_{j=1}^{n}{\left(x_{j}\right)}^{\lambda_{j}}\leq \sum_{j=1}^{n}\lambda_{j}x_{j}.$$

*By Lemma 1, we have the following theorem.*

**Theorem 5.**  *Letting* {*a*_1_,*a*_2_,…,*a_n_*} *be a time series, then*

VGPG (*a*_1_,*a*_2_,…,*a_n_*) ≤ VGPA (*a*_1_,*a*_2_,…,*a_n_*). (22)

## 4. Application in Water, Energy, and Food Efficiency Evaluation

In this section, a case study for the water resource allocation management problem in China is illustrated to show the application of the proposed VGPG operator. Further, comparisons with the existing methods are furnished to show the advantages of the proposed method.

### 4.1. The Case Study

Water resources, energy, and food are the basic resources for human survival, and are also important research topics for sustainable development for the regional economy and ecological environment. With the development of economics in China, the water resources decrease, and the demand for energy increases. Meanwhile, the food production is affected by many factors. It is necessary to study the efficiency among water, energy, and food. In this paper, we will study the comprehensive efficiency of each province from 2007 to 2016. The water/energy-food (W/E-F) coupling efficiencies of 31 provinces from the year 2007 to 2016 in China are listed in Table 1. The W/E-F coupling efficiency means the efficiency of water and energy to food, that is, the input is water and energy, and the output is water. The aggregate values of each province in a period time (such as from 2007 to 2016) could be provided to the people’s central government of China to see the comprehensive efficiency performances. The data are from Zhang and Xu [31]. It is obvious that the data are a time series, and the VG is suitable for dealing with this problem.

In order to compute the comprehensive efficiency of each province, the following steps are involved.

**Figure 3:**
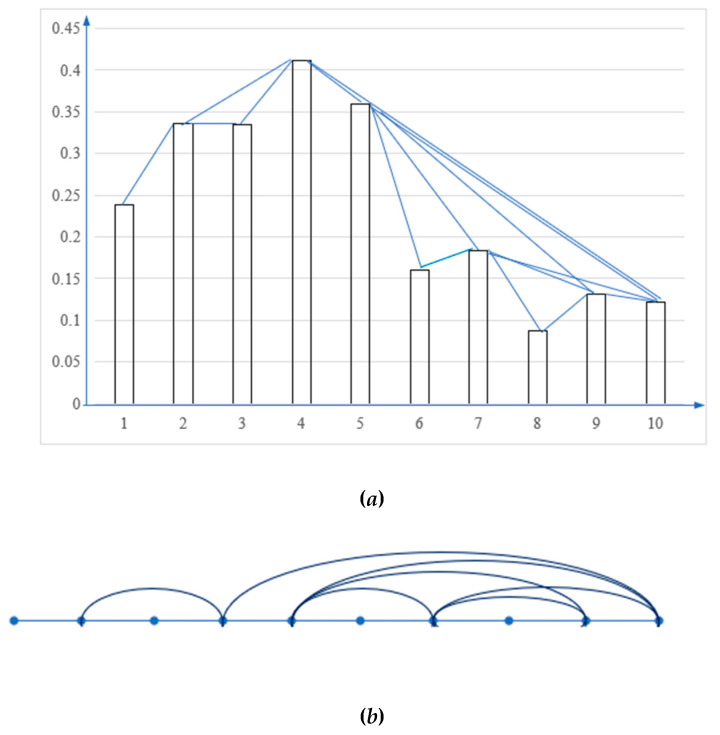
The visibility graph of Beijing. (**a**) the histogram shows a time series with eight data values; (**b**) The visibility rays in the histogram define the links connecting the nodes in the graph.

For the data of Beijing, the VG is shown in Figure 3. The horizontal line (*x* axis) denotes a time series of 10 years, and the vertical line (*y* axis) denotes the efficiencies values for each year in Figure 3a, and Figure 3b is the topology of Figure 3a. By Equation (12), the visibility matrix of Beijing is:

$$V=\left[\begin{array}{cccccccccc} 0 & 1 & 0 & 0 & 0 & 0 & 0 & 0 & 0 & 0\\ 1 & 0 & 1 & 1 & 0 & 0 & 0 & 0 & 0 & 0\\ 0 & 1 & 0 & 1 & 0 & 0 & 0 & 0 & 0 & 0\\ 0 & 1 & 1 & 0 & 1 & 0 & 0 & 0 & 0 & 1\\ 0 & 0 & 0 & 1 & 0 & 1 & 1 & 0 & 1 & 1\\ 0 & 0 & 0 & 0 & 1 & 0 & 1 & 0 & 0 & 0\\ 0 & 0 & 0 & 0 & 1 & 1 & 0 & 1 & 1 & 1\\ 0 & 0 & 0 & 0 & 0 & 0 & 1 & 0 & 1 & 0\\ 0 & 0 & 0 & 0 & 1 & 0 & 1 & 1 & 0 & 1\\ 0 & 0 & 0 & 1 & 1 & 0 & 1 & 0 & 1 & 0 \end{array}\right]$$

By Equations (13) and (14), the support matrix of Beijing is:

$$\mathrm{Sup}=\left[\begin{array}{cccccccccc} 0 & 0.903 & 0 & 0 & 0 & 0 & 0 & 0 & 0 & 0\\ 0.903 & 0 & 0.999 & 0.924 & 0 & 0 & 0 & 0 & 0 & 0\\ 0 & 0.999 & 0 & 0.923 & 0 & 0 & 0 & 0 & 0 & 0\\ 0 & 0.924 & 0.923 & 0 & 0.948 & 0 & 0 & 0 & 0 & 0.71\\ 0 & 0 & 0 & 0.948 & 0 & 0.8 & 0.824 & 0 & 0.772 & 0.762\\ 0 & 0 & 0 & 0 & 0.8 & 0 & 0.976 & 0 & 0 & 0\\ 0 & 0 & 0 & 0 & 0.824 & 0.976 & 0 & 0.903 & 0.948 & 0.938\\ 0 & 0 & 0 & 0 & 0 & 0 & 0.903 & 0 & 0.955 & 0\\ 0 & 0 & 0 & 0 & 0.772 & 0 & 0.948 & 0.955 & 0 & 0.99\\ 0 & 0 & 0 & 0.71 & 0.762 & 0 & 0.938 & 0 & 0.99 & 0 \end{array}\right]$$

By Equations (8), (15), and (16), the weights of different years of Beijing are: *w* = (0.0494, 0.0992, 0.0758, 0.1169, 0.1325, 0.072, 0.145, 0.0741, 0.121, 0.1141).

It shows that the year 2013 has the largest weight, and the year 2011 has the second largest weight, although these two years have the same visibilities. Similarly, we can obtain the weights for other provinces, which are depicted in Table 2. Then, by Equation (17), we can obtain the VGPG aggregated value of Beijing, which is 0.2135. The weights of OWA are shown in Table 3. In the VGPA aggregation process, we also adopt Equation (13) as the support function.The aggregated values of the other provinces can be obtained in the same way, which are listed in Table 4. From Table 4, we can see that Shanxi has the largest W/E-F coupling efficiency and ranks first, and then Xinjiang is second, and so on. From Table 1, we also know that, although Tianjing has the seven times largest efficiency, the efficiencies in 2014, 2015, and 2016 are smaller; thus, in Table 4, its overall VGPG aggregated efficiency ranks fourth. It is similar for Qinghai.

### 4.2. Comparative Analysis

**Figure 4:**
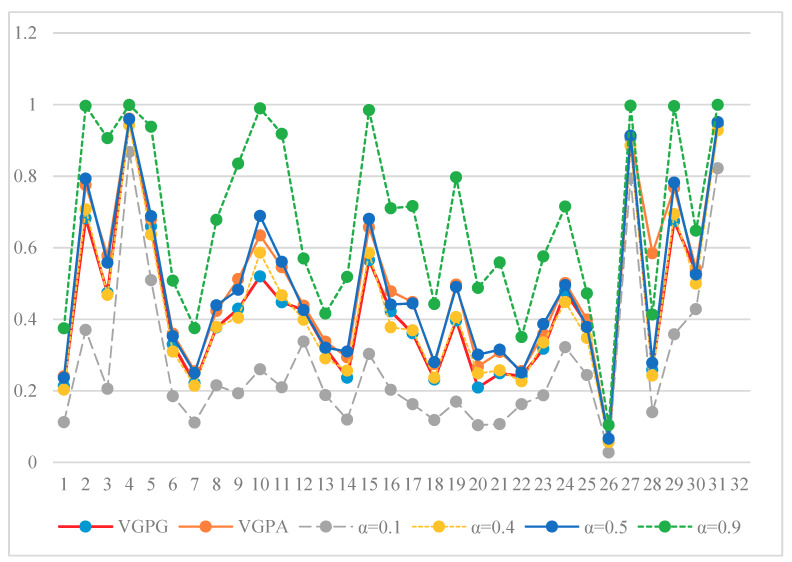
The aggregated results by different operators.

In order to show the performances of the proposed method, we provide the comparisons with the existing methods. We also provide the VGPA values, and the OWA aggregation values by different orness degrees *α* (*α* = 0.1,0.4,0.5,0.9). The weights of OWA are shown in Table 3. In the VGPA aggregation process, we also adopt Equation (13) as the support function. One reason is that the support function proposed by Jiang et al. [30] does not satisfy the third property as verified in Remark 1, and another is to compare the performances with the proposed VGPG operator under the condition that they have the same weights. All the aggregated values by these different operators are listed in Table 4 and described in Figure 4.

From Table 4, we can see that Shanxi ranks first, Xinjiang ranks second except for *α* = 0.9, and Shanxi ranks third for all the operators. Tianjin ranks fourth, Qinghai ranks fifth except for *α* = 0.1, and Xizang ranks last for all the operators. Other rankings fluctuate by different operators.

From Figure 4, it is obvious that the values are very different when the *α* are different. Specifically, the aggregation values of each alternative increase when the value of *α* increases. For example, the OWA aggregation value of Beijing is 0.1126 when *α* = 0.1, 0.2033 when *α* = 0.4, 0.2367 when *α* = 0.5, and 0.3751 when *α* = 0.9. This also happens for the other alternatives. What is more, we can find that the weights increase steadily when *α* < 0.5 from Table 4, and the weight of last time is larger than 0.5. Because the OWA aggregation first reorders the input arguments in descending order, and the largest input argument has the lowest weight and the smallest input argument has the largest weight, that is why it has the smallest OWA aggregation value when *α* = 0.1. On the contrary, the weights decrease when *α* > 0.5, and it have the largest OWA aggregation value when *α* = 0.9. Further, the OWA aggregation will become the average aggregation operator as all the weights have the same importance (*w_i_* = 1/*n*). Overall, the weights are determined when *α* is specified. This shows that the weights of OWA obtained by the maximization of the entropy and orness degree show a lack of objectivity. The relationship of input argument information is not well considered. As the weights of OWA obtained by Equation (2) are in ascending or descending order, it is difficult for the decision maker to choose the best value of *α*.

For the VGPG operator and VGPA operator, it shows that all the VGPG aggregation values are smaller than VGPA aggregation values, and this verifies Theorem 5. All the aggregation values of VGPG and VGPA lie between the results of the OWA operator when *α* = 0.1 and *α* = 0.9. More specifically, the results of the VGPG operator are smaller than the results of the OWA operator when *α* = 0.5, and the results of the VGPA operator are larger than the results of the OWA operator when *α* = 0.4. For the VGPG and VGPA operators, the polyline charts are almost consistent with the polyline chart of OWA aggregation when *α* = 0.5. The change tendency of the three operators is almost same, which also shows that the proposed VGPG operator is correct and effective. However, the weights of the VGPG operator are objective as they are data driven, i.e., the weights of the VGPG operator not only consider the visibility, but also the similarity (or support) with the connected values.

## 5. Conclusions

In the real decision-making process, there are so many time series values that need to be aggregated. In this paper, motivated by the PG operator and the visibility graph, we develop a VGPG operator. First of all, all the time series data can be transformed into a VG. Then, according to the links among different nodes, the visibility matrix can be obtained. According to the visibility of two nodes, we design a new support function between two values. Then, all the support degrees from others of one value can be obtained. Finally, the overall value of each alternative at different times can be aggregated by the developed VGPG operator. Further, some properties of the proposed operator, such as boundness and idempotency, are investigated. Finally, a real application for water, energy and food coupling efficiency evaluation in China is illustrated, and comparisons with the existing methods are also furnished to show the feasibility and advantages of the proposed method.

Compared with the existing method, the proposed method has the following advantages:

(1) The time series data are transformed into a VG, and a visibility matrix is developed to denote the links of different data, while the other methods do not consider the relationships of different data.
(2) The support function is developed to measure the similarity of two linked values, while the power aggregation measures the similarity between any two values. It does not consider whether the two values are linked.
(3) The weights determined by the VG and support function are more objective and reasonable, while the weights of OWA obtained by various methods are stationary when the parameters are specified. These methods do not consider the relationship of the input arguments.

In the future, we will explore the proposed aggregation operator in other areas, such as economics [32], weather forecast renewable energy sources [33], and so forth. Another prominent interesting future work is to propose the VG-POWA, VG-POWG operators, and this extends to other types of values, such as the intuitionistic fuzzy set [34,35,36], interval-valued intuitionistic fuzzy set [37], Pythagorean fuzzy group decision making [38], hesitant fuzzy set [39,40,41], neutrosophic information [42], etc.

## Figures and Tables

**Table 1:** The 2007–2016 W/E-F coupling efficiency value of each region.

Province	2007	2008	2009	2010	2011	2012	2013	2014	2015	2016
Beijing	0.239	0.336	0.335	0.412	0.36	0.16	0.184	0.087	0.132	0.122
Tianjin	1	1	1	1	1	1	1	0.277	0.308	0.348
Hebei	0.726	0.672	0.987	0.761	0.917	0.501	0.517	0.174	0.17	0.159
Shanxi	1	1	1	0.817	0.941	0.896	0.942	1	1	1
InnerMongolia	0.546	0.584	0.554	0.462	0.593	0.642	0.604	0.946	0.991	0.961
Liaoning	0.517	0.527	0.48	0.359	0.497	0.298	0.32	0.155	0.182	0.194
Jilin	0.39	0.358	0.36	0.245	0.383	0.282	0.18	0.105	0.102	0.094
HeiLongJiang	0.728	0.663	0.655	0.493	0.571	0.373	0.31	0.206	0.206	0.189
Shanghai	0.607	0.693	0.551	0.674	1	0.371	0.42	0.176	0.175	0.157
Jiangsu	1	1	1	1	1	0.713	0.524	0.209	0.204	0.242
Zhejiang	0.597	0.79	0.907	0.966	0.939	0.427	0.423	0.162	0.2	0.197
Anhui	0.324	0.438	0.582	0.487	0.615	0.403	0.353	0.348	0.365	0.346
Fujian	0.399	0.379	0.399	0.348	0.438	0.346	0.387	0.164	0.174	0.175
Jiangxi	0.511	0.558	0.395	0.472	0.48	0.183	0.161	0.126	0.099	0.115
Shandong	1	1	1	0.961	0.963	0.541	0.549	0.267	0.264	0.262
Henan	0.583	0.544	0.577	0.695	0.788	0.314	0.293	0.267	0.193	0.161
Hebei	0.633	0.758	0.69	0.665	0.706	0.279	0.268	0.174	0.14	0.132
Hunan	0.39	0.374	0.476	0.424	0.426	0.2	0.176	0.132	0.105	0.099
Guangdong	0.763	0.757	0.697	0.669	0.86	0.453	0.281	0.144	0.144	0.138
Guangxi	0.345	0.505	0.501	0.484	0.467	0.261	0.181	0.101	0.085	0.084
Hainan	0.618	0.561	0.449	0.467	0.36	0.197	0.215	0.084	0.096	0.106
Chongqing	0.289	0.372	0.341	0.302	0.343	0.182	0.198	0.15	0.166	0.169
Sichuan	0.523	0.592	0.606	0.49	0.484	0.382	0.274	0.164	0.167	0.188
Guizhou	0.677	0.668	0.788	0.573	0.426	0.433	0.271	0.392	0.362	0.368
Yunnan	0.388	0.481	0.46	0.389	0.445	0.448	0.48	0.228	0.219	0.248
Xizang	0.118	0.097	0.088	0.07	0.083	0.067	0.052	0.019	0.025	0.044
Shaanxi	0.77	0.781	0.897	0.829	0.857	1	1	1	1	1
Gansu	0.438	0.436	0.323	0.251	0.351	0.334	0.273	0.124	0.126	0.124
Qinghai	0.897	1	1	1	1	1	1	0.363	0.269	0.292
Ningxia	0.524	0.566	0.492	0.471	0.595	0.603	0.704	0.426	0.407	0.468
Xinjiang	0.929	1	1	0.763	0.819	1	1	1	1	1

**Table 2:** The weights of the proposed method.

Province	*w*_1_	*w*_2_	*w*_3_	*w*_4_	*w*_5_	*w*_6_	*w*_7_	*w*_8_	*w*_9_	*w*_10_
Beijing	0.0494	0.0992	0.0758	0.1169	0.1325	0.072	0.145	0.0741	0.121	0.1141
Tianjin	0.0676	0.1014	0.1014	0.1014	0.1014	0.1014	0.0991	0.1073	0.1094	0.1094
Hebei	0.0813	0.0796	0.125	0.0792	0.1348	0.0777	0.1373	0.0803	0.1178	0.087
Shanxi	0.0519	0.0779	0.148	0.0699	0.1483	0.0988	0.1256	0.15	0.0779	0.0519
InnerMongolia	0.0634	0.1362	0.1326	0.0687	0.1341	0.1352	0.0649	0.1436	0.0724	0.0488
Liaoning	0.052	0.1022	0.0996	0.0716	0.1781	0.0725	0.1402	0.0733	0.1177	0.0929
Jilin	0.0826	0.0829	0.1015	0.0578	0.1652	0.1104	0.098	0.0939	0.1146	0.0931
HeiLongJiang	0.0675	0.069	0.1102	0.0651	0.1478	0.1035	0.1261	0.0833	0.1261	0.1013
Shanghai	0.0715	0.1261	0.0932	0.1001	0.1368	0.0658	0.131	0.0831	0.1109	0.0815
Jiangsu	0.0571	0.0856	0.0856	0.0856	0.1051	0.1011	0.1261	0.1041	0.1236	0.1261
Zhejiang	0.0589	0.0877	0.092	0.095	0.1129	0.081	0.1555	0.088	0.1303	0.0987
Anhui	0.0633	0.066	0.1076	0.0668	0.1584	0.1118	0.1124	0.1121	0.1363	0.0653
Fujian	0.1043	0.0783	0.1294	0.0756	0.1505	0.0759	0.1392	0.0732	0.0999	0.0737
Jiangxi	0.0453	0.1074	0.0641	0.0889	0.1439	0.0839	0.1281	0.1062	0.1048	0.1275
Shandong	0.0561	0.0842	0.1101	0.0831	0.1326	0.0721	0.1326	0.1041	0.1126	0.1124
Henan	0.1158	0.1132	0.0959	0.113	0.1434	0.0625	0.0863	0.1067	0.0824	0.0808
Hebei	0.0455	0.1132	0.0945	0.0933	0.1543	0.0622	0.1261	0.1044	0.1035	0.103
Hunan	0.069	0.0686	0.1122	0.0702	0.1555	0.0655	0.1318	0.1096	0.109	0.1085
Guangdong	0.0671	0.1098	0.0868	0.0855	0.146	0.1039	0.1119	0.0889	0.1119	0.0883
Guangxi	0.045	0.0694	0.0729	0.0726	0.1311	0.1067	0.1311	0.1106	0.1304	0.1303
Hainan	0.0657	0.0879	0.0675	0.1564	0.1192	0.0663	0.1465	0.0672	0.1229	0.1004
Chongqing	0.0461	0.0928	0.0945	0.0703	0.1944	0.0679	0.1379	0.0901	0.1148	0.0913
Sichuan	0.0467	0.0706	0.1047	0.0696	0.1247	0.106	0.111	0.0934	0.1291	0.1442
Guizhou	0.0619	0.0618	0.1419	0.1269	0.0749	0.134	0.0584	0.1335	0.0931	0.1136
Yunnan	0.0419	0.1277	0.1287	0.0631	0.1286	0.108	0.1574	0.0817	0.0813	0.0817
Xizang	0.0915	0.0748	0.0934	0.0561	0.1662	0.0927	0.1113	0.0744	0.1106	0.1288
Shaanxi	0.0832	0.0836	0.1613	0.1086	0.1102	0.1333	0.0872	0.0872	0.0872	0.0582
Gansu	0.051	0.1408	0.0967	0.093	0.1218	0.1177	0.1148	0.0728	0.0983	0.0929
Qinghai	0.0648	0.099	0.1025	0.1025	0.1025	0.1025	0.0999	0.1093	0.1077	0.1093
Ningxia	0.0466	0.1338	0.1092	0.0895	0.1339	0.0688	0.1578	0.0871	0.0862	0.0871
Xinjiang	0.0601	0.0913	0.1429	0.1082	0.1117	0.1429	0.0935	0.0935	0.0935	0.0624

**Table 3:** The OWA weights.

	*w*_1_	*w*_2_	*w*_3_	*w*_4_	*w*_5_	*w*_6_	*w*_7_	*w*_8_	*w*_9_	*w*_10_
*α* = 0.1	0.0007	0.0014	0.0029	0.0061	0.0127	0.0268	0.0563	0.1186	0.2495	0.5250
*α* = 0.4	0.0576	0.0644	0.0720	0.0804	0.0899	0.1005	0.1123	0.1256	0.1403	0.1569
*α* = 0.5	0.1	0.1	0.1	0.1	0.1	0.1	0.1	0.1	0.1	0.1
*α* = 0.9	0.5250	0.2495	0.1186	0.0563	0.0267	0.0127	0.0061	0.0029	0.0014	0.0007

**Table 4:** The aggregation results by different operators.

Province	VGPG	Rank	VGPA	Rank	*α* = 0.1	Rank	*α* = 0.4	Rank	*α* = 0.5	Rank	*α* = 0.9	Rank
Beijing	0.2135	29	0.2409	30	0.1126	27	0.2033	30	0.2367	30	0.3751	29
Tianjin	0.6824	4	0.7753	4	0.3704	6	0.7075	4	0.7933	4	0.9965	4
Hebei	0.4743	11	0.5775	9	0.2057	15	0.4684	10	0.5584	10	0.9065	10
Shanxi	0.9594	1	0.9609	1	0.8677	1	0.9427	1	0.9596	1	0.9991	2
InnerMongolia	0.6595	6	0.6794	6	0.5096	4	0.6369	6	0.6883	7	0.9383	8
Liaoning	0.3303	20	0.3600	20	0.1852	20	0.3101	21	0.3529	21	0.5080	22
Jilin	0.2214	28	0.2549	29	0.1116	28	0.2143	29	0.2499	29	0.3754	28
HeiLongJiang	0.3773	18	0.4236	18	0.2156	13	0.3787	16	0.4394	17	0.6783	16
Shanghai	0.4298	13	0.5126	12	0.1934	17	0.4043	14	0.4824	14	0.8352	11
Jiangsu	0.52	9	0.6347	8	0.2604	11	0.5865	7	0.6892	6	0.9895	6
Zhejiang	0.4478	12	0.5458	10	0.2102	14	0.4672	11	0.5608	9	0.9184	9
Anhui	0.4262	14	0.4381	17	0.3378	8	0.3988	15	0.4261	18	0.5698	19
Fujian	0.3189	21	0.3376	22	0.1883	18	0.2908	22	0.3209	22	0.4161	26
Jiangxi	0.2371	26	0.2938	24	0.1200	25	0.2570	24	0.3100	24	0.5184	21
Shandong	0.5654	7	0.6568	7	0.3033	10	0.5854	8	0.6807	8	0.9846	7
Henan	0.4224	15	0.4783	15	0.2029	16	0.3778	17	0.4415	16	0.7102	15
Hebei	0.3609	19	0.4482	16	0.1630	22	0.3695	18	0.4445	15	0.7162	13
Hunan	0.2318	27	0.275	26	0.1184	26	0.2371	27	0.2802	26	0.4426	25
Guangdong	0.3967	16	0.4972	14	0.1700	21	0.4064	13	0.4906	13	0.7969	12
Guangxi	0.2095	30	0.2682	27	0.1042	30	0.2491	25	0.3014	25	0.4879	23
Hainan	0.2491	24	0.3088	23	0.1074	29	0.2575	23	0.3153	23	0.5591	20
Chongqing	0.2414	25	0.2556	28	0.1630	23	0.2267	28	0.2512	28	0.3505	30
Sichuan	0.3177	22	0.359	21	0.1876	19	0.3355	20	0.3870	19	0.5758	18
Guizhou	0.4775	10	0.5013	13	0.3222	9	0.4484	12	0.4958	12	0.7152	14
Yunnan	0.384	17	0.3993	19	0.2443	12	0.3479	19	0.3786	20	0.4724	24
Xizang	0.0581	31	0.0659	31	0.0280	31	0.0569	31	0.0663	31	0.1045	31
Shaanxi	0.9073	3	0.9116	3	0.7934	3	0.8852	3	0.9134	3	0.9968	3
Gansu	0.2587	23	0.5847	25	0.1405	24	0.2428	26	0.2780	27	0.4134	27
Qinghai	0.6745	5	0.7676	5	0.3582	7	0.6943	5	0.7821	5	0.9959	5
Ningxia	0.5332	8	0.5412	11	0.4283	5	0.4996	9	0.5256	11	0.6478	17
Xinjiang	0.9455	2	0.9499	2	0.8220	2	0.9284	2	0.9511	2	0.9993	1
